# Management of Mirizzi Syndrome Type I: A Case Report

**DOI:** 10.7759/cureus.80799

**Published:** 2025-03-18

**Authors:** Shirly S, Mathesh A, Suismitha S, Mohamed Aqeel A, Dhivya K

**Affiliations:** 1 Department of Pharmacy Practice, C.L. Baid Metha College of Pharmacy, Chennai, IND

**Keywords:** acute cholangitis, ercp, gallstones, mirizzi syndrome, mrcp

## Abstract

Mirizzi syndrome is a rare yet clinically significant condition characterized by mechanical obstruction of the common bile duct (CBD) and the hepatic duct. This obstruction arises from external compression caused by one or more impacted gallstones, most often lodged in Hartmann’s pouch, a segment of the gallbladder located near the cystic duct. The prolonged presence of these gallstones can lead to localized inflammation, fibrosis, and scarring, further exacerbating the narrowing of the bile ducts. This condition often presents with symptoms resembling other biliary disorders, making its diagnosis challenging. This report presents a rare case of Mirizzi syndrome Type I in a 64-year-old male patient. The patient was admitted with complaints of right upper quadrant pain for the past two days, radiating to the back, epigastric region, and left hypochondrium. A comprehensive diagnostic evaluation, including magnetic resonance cholangiopancreatography (MRCP) and endoscopic retrograde cholangiopancreatography (ERCP), confirmed the diagnosis of Mirizzi syndrome Type I. The patient underwent ERCP with precut sphincterotomy, biliary plastic stent placement, and cholecystectomy.

## Introduction

Mirizzi syndrome, initially observed in the early 1900s, was officially named in 1948 in honor of physician Pablo Luis Mirizzi. Mirizzi syndrome is an uncommon but clinically significant condition resulting from mechanical obstruction of the common bile duct (CBD) and hepatic duct due to external compression by impacted gallstones, typically located in Hartmann’s pouch near the cystic duct [1]. This rare complication of cholelithiasis occurs globally in 0.05%-0.3% of the population and is more common in females aged 53-70 years [2]. Chronic compression often leads to inflammation, scarring, or fibrosis of the surrounding bile ducts, adding to the complexity of diagnosis and management. Mirizzi syndrome is classified into five types based on the extent of bile duct involvement. Type I, the mildest form, is defined by the absence of a fistula between the gallbladder and bile duct. Types II to IV involve varying degrees of bile duct erosion, while Type V is associated with a cholecystoenteric fistula [3]. Symptoms typically include right upper quadrant pain, jaundice, fever, nausea, and dark urine, mimicking other hepatobiliary conditions. Management is tailored according to the severity and type of the condition [4]. For Type I, laparoscopic or open cholecystectomy is often the preferred treatment, sometimes with bile duct drainage via a T-tube. Prompt diagnosis and personalized treatment are essential to prevent complications such as cholangitis and bile duct injury [5]. This report discusses a case of a 64-year-old male diagnosed with Mirizzi syndrome Type I.

## Case presentation

A 64-year-old male presented with complaints of right upper quadrant pain for the past two days, radiating to the back, epigastric region, and left hypochondrium. The pain was intermittent and episodic, lasting one to two hours, with increasing severity over the past week. He reported yellowish discoloration of the eyes and urine for one week, which progressively deepened in intensity, accompanied by intermittent high-grade fever with chills requiring antipyretics. There was no history of pruritus, pale stools, decreased urine output, prior episodes of jaundice, or any previous laparoscopic surgeries or endoscopic interventions. On examination, he was conscious, oriented, and afebrile, with a blood pressure of 150/70 mmHg, a pulse rate of 84 beats per minute, and SpO₂ of 100%. Cardiovascular and respiratory system examinations were unremarkable, and the abdomen was soft and non-tender. There was no evidence of Murphy's sign. He was moving all four limbs without difficulty. The laboratory findings revealed several abnormalities, which are shown in Table 1.

**Table 1:** Preoperative Laboratory Investigations

Parameter	Observed Value	Normal Range
Haemoglobin (Hb)	10.7 g/dL	14-18 g/dL
Packed cell volume (PCV)	13.1%	38-54%
White blood cell count (WBC)	19,311 cells/mm³	4,000-11,000 cells/mm³
Red blood cell count (RBC)	4.0 million/mm³	4.5-6 million/mm³
Red cell distribution width (RDW-CV)	14.9%	11.5-14.5%
Erythrocyte sedimentation rate (ESR)	84 mm/hr	<20 mm/hr
Lymphocytes	9.6%	20-40%
Eosinophils	0.1%	1-6%
Neutrophils	8.55%	40-70%
Total bilirubin	12.39 mg/dL	0.2-1.2 mg/dL
Direct bilirubin	11.41 mg/dL	0.2-0.6 mg/dL
Alkaline phosphatase (ALP)	185 U/L	40-130 U/L
Alanine transaminase (ALT)	81 U/L	7-56 U/L
Aspartate transaminase (AST)	57 U/L	5-40 U/L
Gamma-glutamyl transferase (GGT)	199 U/L	9-48 U/L
Albumin/globulin (A/G) ratio	1	>1
Sodium	131 mmol/L	135-145 mmol/L

Chronic bile duct inflammation caused a reduction in the patient's hemoglobin (Hb), packed cell volume (PCV), and red blood cell (RBC) count, indirectly contributing to anemia. An elevated white blood cell (WBC) count and red cell distribution width (RDW-CV) suggested an active infection, with an elevated erythrocyte sedimentation rate (ESR) indicating the presence of a severe infection. The lymphocyte, eosinophil, and neutrophil counts were low, likely reflecting the immune system's response to the infection. Liver function tests showed elevated total and direct bilirubin levels, indicating biliary obstruction. Liver enzymes, such as alkaline phosphatase (ALP), alanine aminotransferase (ALT), aspartate aminotransferase (AST), and gamma-glutamyl transferase (GGT), were markedly elevated due to cholestasis and liver cell damage. Additionally, the albumin-to-globulin (A/G) ratio was low, likely due to chronic illness and inflammation, which led to a decrease in albumin and an increase in globulin levels as part of immune activation. Renal function tests revealed electrolyte imbalances, with low sodium levels being particularly notable.

**Figure 1:**
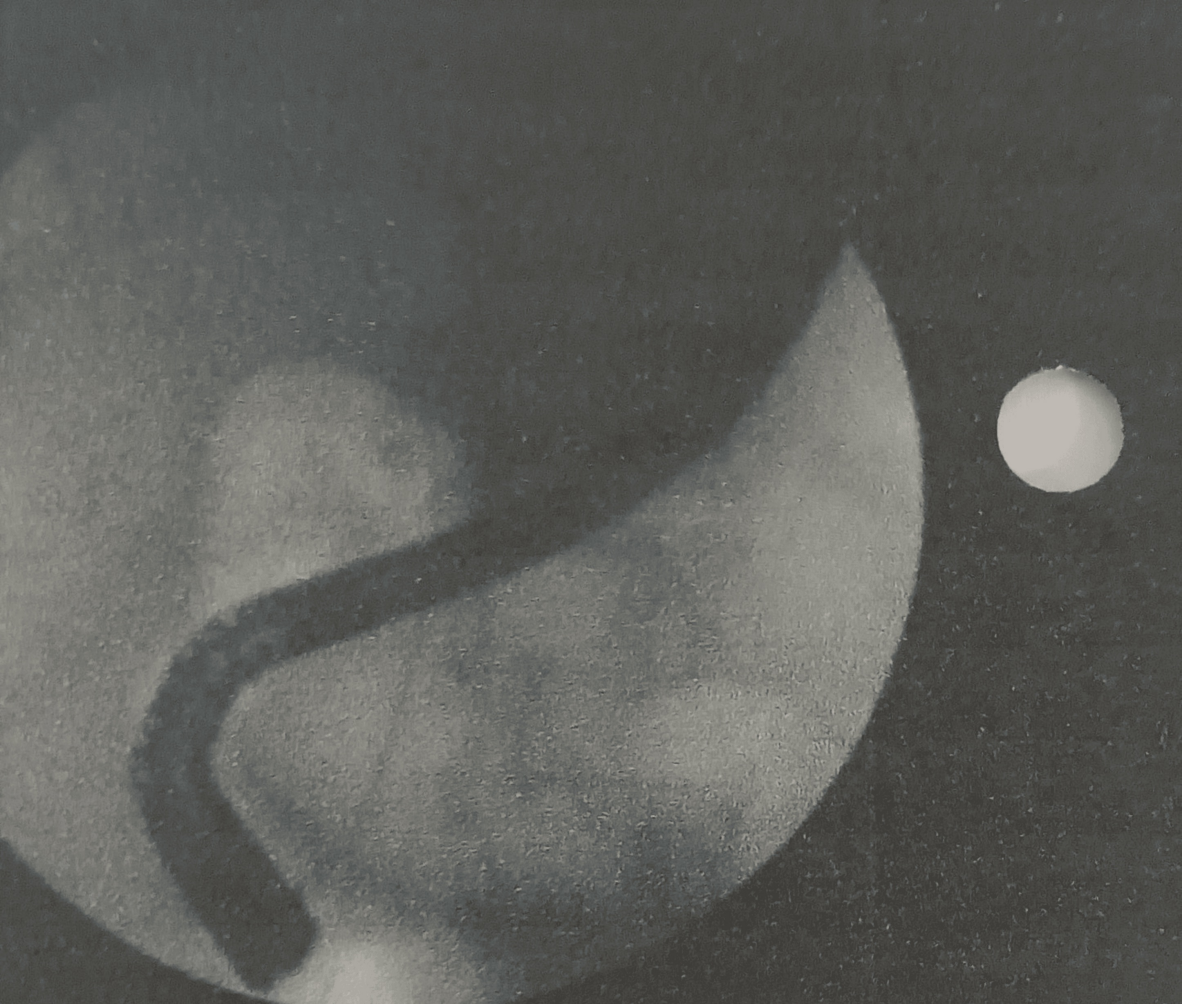
Extrinsic Compression of the Common Bile Duct

**Figure 2:**
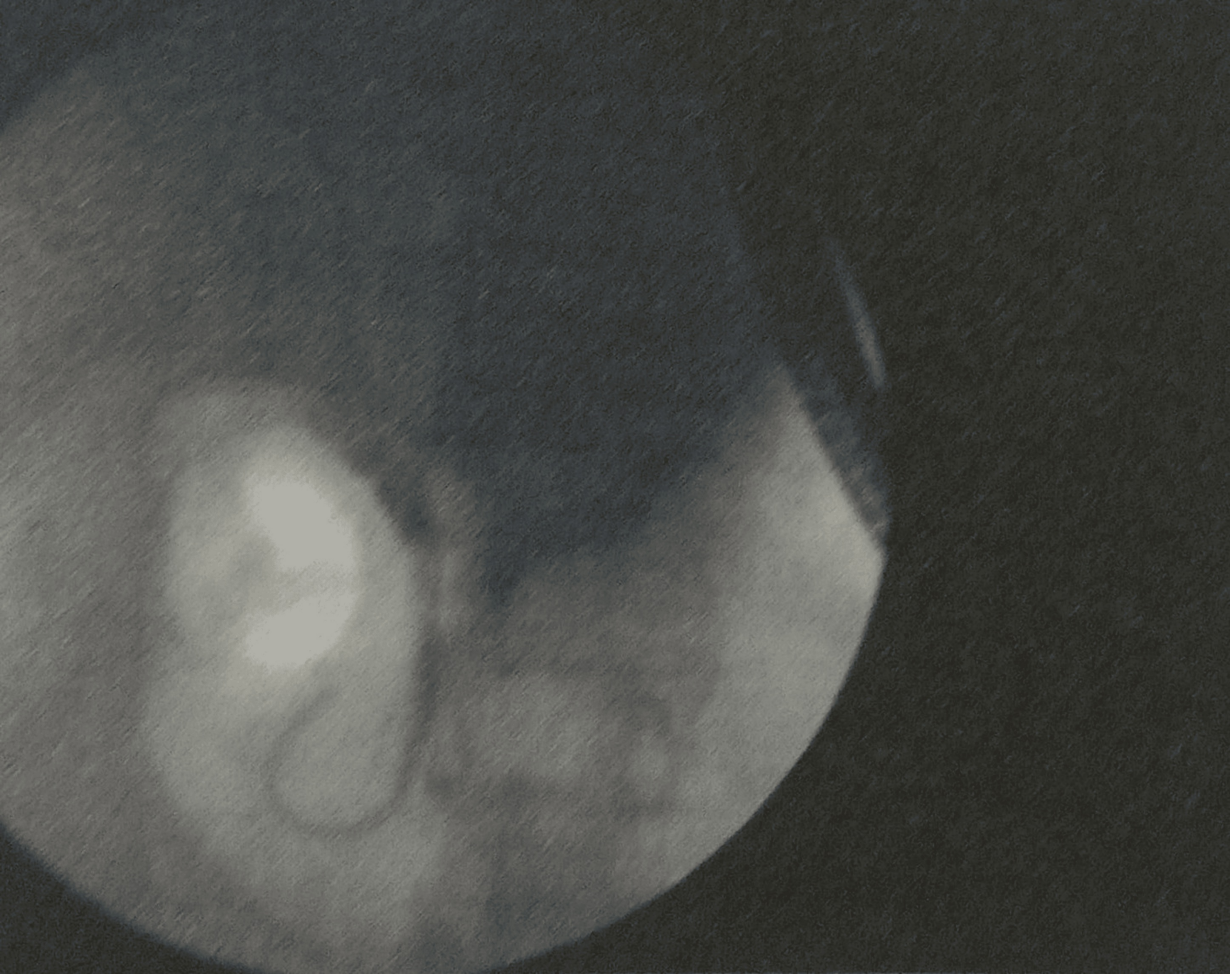
Intrahepatic Bile Duct Dilation With Proximal Obstruction

**Figure 3:**
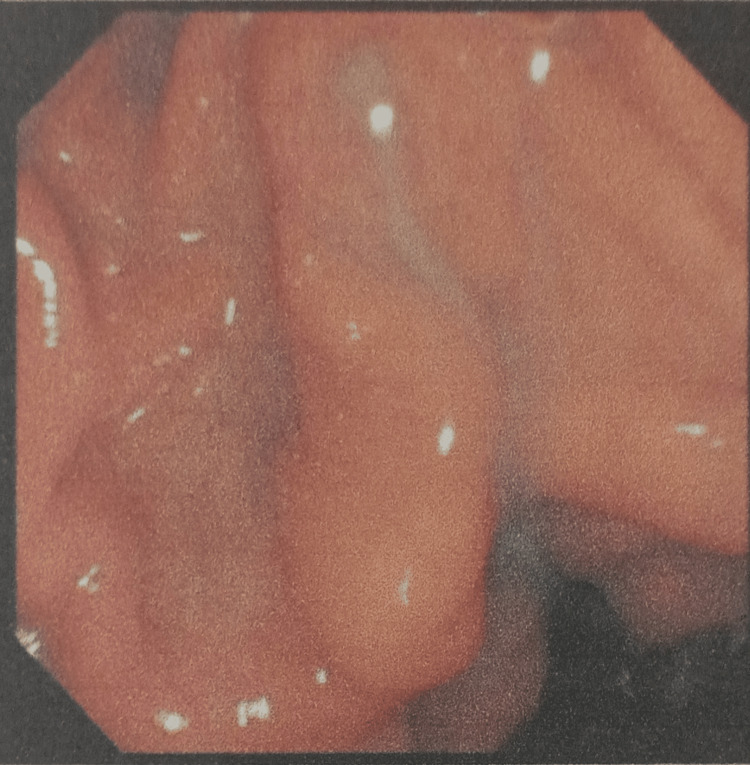
Inflamed Common Bile Duct Wall Due to Acute Cholangitis

**Figure 4:**
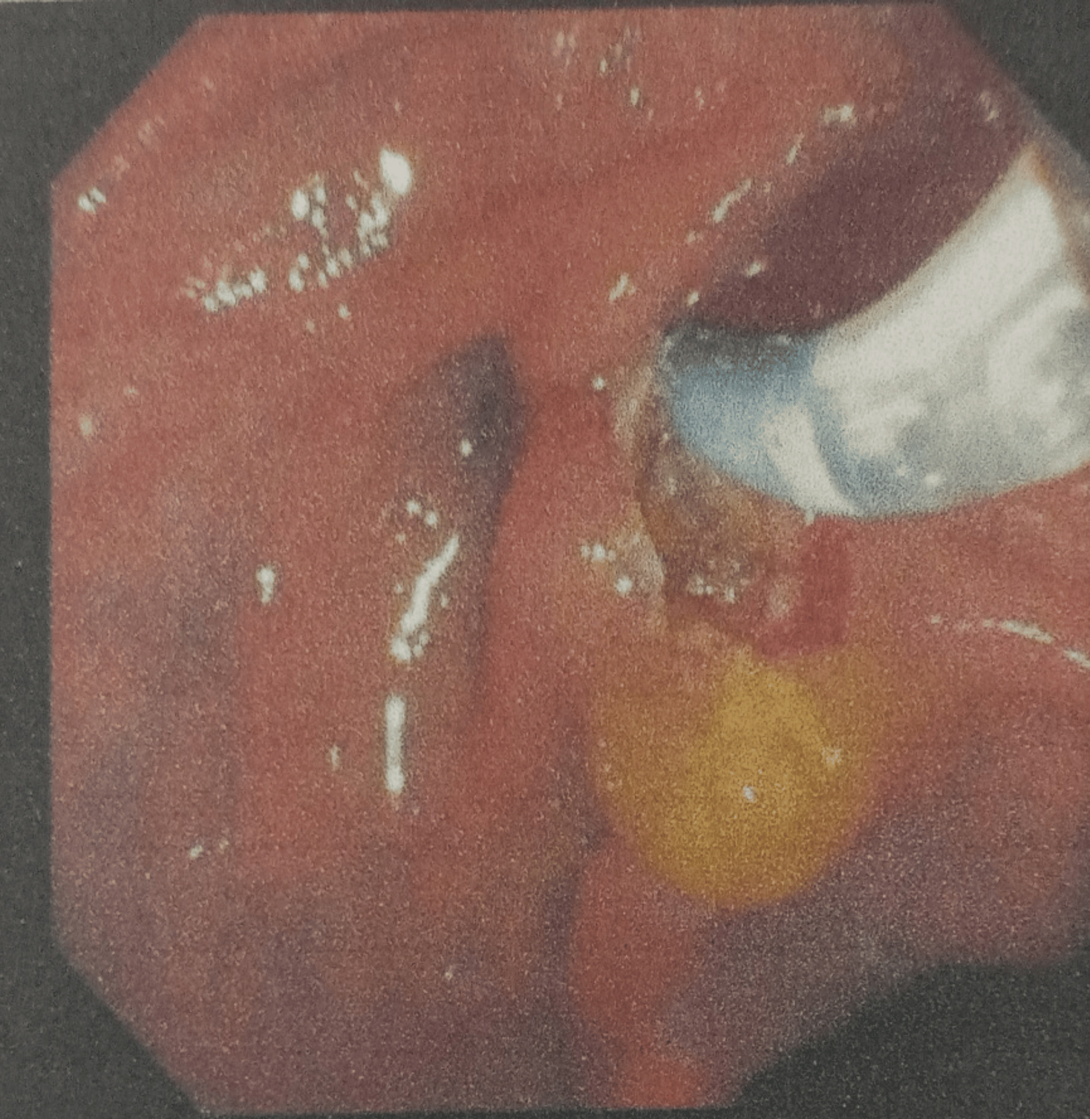
Obstructive Calculus in the Proximal Common Bile Duct

**Figure 5:**
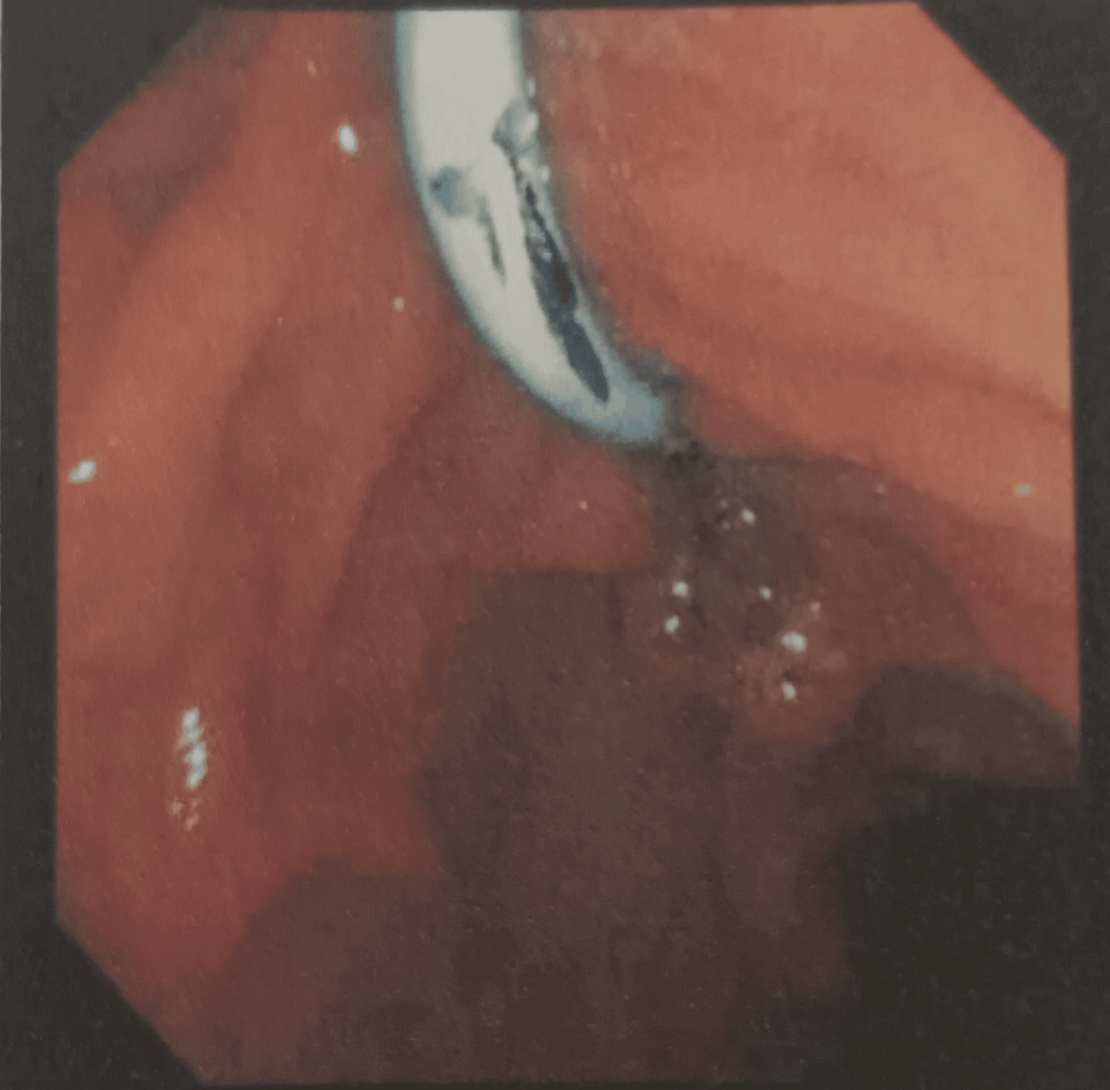
Plastic Biliary Stent Placement Post-ERCP ERCP: endoscopic retrograde cholangiopancreatography.

Magnetic resonance cholangiopancreatography (MRCP) showed that the liver and pancreas were normal. The CBD was dilated to approximately 10 mm, with a filling defect measuring around 9.1 × 8.5 mm in the proximal CBD, located about 5 cm from the ampulla, consistent with a calculus. This was associated with upstream dilation. The CBD, common hepatic duct, and mild intrahepatic biliary radical dilation were noted. The gallbladder was partially distended and contained sludge. These findings were consistent with acute cholangitis and Mirizzi syndrome Type I. Endoscopic retrograde cholangiopancreatography (ERCP) with precut sphincterotomy, biliary plastic stent placement, and cholecystectomy were planned and performed. Following surgery, the patient was administered piperacillin-tazobactam 4.5 g, pantoprazole 40 mg, paracetamol 1 g, vitamin K 10 mg, and liquid paraffin with milk of magnesia. After two days of treatment, the patient’s condition had stabilized, and he was discharged. Table 2 shows the daily progression of the patient’s health condition, indicating improvements in the blood profile, and reductions in ESR, bilirubin, and liver enzyme levels. The MRCP report is shown in Figures 1 and 2. Figure 1 depicts the extrinsic compression of the bile duct, a hallmark feature of Mirizzi syndrome. Figure 2 represents intrahepatic bile duct dilation, suggesting proximal obstruction that correlates with extrinsic compression. ERCP reports are shown in Figures 3-5. Figure 3 shows the inflamed wall of the CBD, likely attributed to acute cholangitis, typically due to biliary obstruction caused by gallstones. Figure 4 displays a gallstone (calculus) obstructing the proximal part of the CBD, which likely contributes to the inflammation seen in acute cholangitis. Figure 5 illustrates the insertion of a plastic biliary stent in the CBD following the ERCP procedure, aiding in relieving the obstruction by restoring bile flow and reducing pressure.

**Table 2:** Post-operative Laboratory Investigations A/G ratio: albumin/globulin ratio, ALP: alkaline phosphatase, ALT: alanine aminotransferase, AST: aspartate aminotransferase, ESR: erythrocyte sedimentation rate, GGT: gamma-glutamyl transferase, Hb: hemoglobin, PCV: packed cell volume, RDW-CV: red cell distribution width, RBC: red blood cell count, WBC: white blood cell count.

Parameter	Day 1	Day 2	Normal Range
Hb	11 g/dL	12 g/dL	14-18 g/dL
PCV	37%	40%	38-54%
WBC	12,311 cells/mm³	10,000 cells/mm^3^	4,000-11,000 cells/mm³
RBC	4.4 million/mm³	4.7 million/mm^3^	4.5-6 million/mm³
RDW-CV	14.9%	14%	11.5-14.5%
ESR	25 mm/hour	15mm/hour	<20 mm/hour
Lymphocytes	20%	25%	20-40%
Eosinophils	2%	2%	1-6%
Neutrophils	40%	45%	40-70%
Total bilirubin	1.5 mg/dL	0.4 mg/dL	0.2-1.2 mg/dL
Direct bilirubin	0.8 mg/dL	0.3 mg/dL	0.2-0.6 mg/dL
ALP	129 U/L	120 U/L	40-130 U/L
ALT	60 U/L	50 U/L	7-56 U/L
AST	45 U/L	30 U/L	5-40 U/L
GGT	50 U/L	10 U/L	9-48 U/L
A/G ratio	1	0.1	>1
Sodium	131 mmol/L	136 mmol/L	135-145 mmol/L

## Discussion

Mirizzi syndrome is a condition in which the common hepatic duct becomes obstructed due to a stone lodged in the gallbladder infundibulum or cystic duct. According to Csendes et al., it is classified into five types [6]. The incidence of Mirizzi syndrome differs by region, with reported rates of 5.7% in Chile and 4.7% in Mexico. It primarily affects individuals aged 53 to 70 years, with a strong female predominance, accounting for 70% of cases. It is estimated to occur in 0.5% of patients with gallstones and in 0.7% to 25% of patients undergoing cholecystectomy, emphasizing its rarity and diagnostic challenges. Type I primarily results from external compression of the bile duct by the stone, with an incidence rate of 10%-52% [7].

Common symptoms of Mirizzi syndrome include fever, nausea, vomiting, dark urine, right upper quadrant or epigastric pain, and anorexia, often mimicking other biliary or hepatic conditions [8]. According to Won et al., patients often present with symptoms such as upper back and abdominal pain, epigastric pain, jaundice, localized right upper quadrant tenderness, and a positive Murphy’s sign [9]. In this case report, the patient presented with right upper quadrant pain for two days, radiating to the back, epigastric region, and left hypochondrium. The pain was intermittent and episodic, lasting 1-2 hours, and had progressively worsened over the past week. He also reported yellowing of the eyes and urine, along with episodes of high-grade fever and chills. Grohol et al. reported that laboratory findings in these cases may show normal levels of WBC, Hb, hematocrit, electrolytes, and troponin, while liver function tests typically reveal abnormalities in total bilirubin, ALP, ALT, and AST [10], findings that partly align with this case. The laboratory reports of this patient showed abnormalities in the complete blood profile and liver function tests.

Borz-Baba et al. highlighted the importance of MRCP and ERCP as confirmatory diagnostic procedures for Mirizzi syndrome [11]. In terms of treatment, Momah et al. reported the successful placement of a stent in the CBD, while Khokhar et al. recommended cholecystectomy as an effective surgical intervention [12]. In this case, Mirizzi syndrome was confirmed using MRCP and ERCP. The patient then underwent ERCP with precut sphincterotomy and biliary plastic stent placement, followed by cholecystectomy. This complies with the Standard Treatment Guidelines issued by the Ministry of Health and Family Welfare, Government of India, which recommend ERCP accompanied by papillotomy and stenting as a viable treatment for Mirizzi syndrome. During hospitalization, the patient was administered intravenous antibiotics, proton pump inhibitors, antipyretics, vitamin K, and syrup lactulose to address associated symptoms and stabilize his condition. This case report has a few limitations, including its focus on a single patient, limiting generalizability. The lack of long-term follow-up prevents assessment of potential complications or recurrence of Mirizzi syndrome.

## Conclusions

We present a case of a 64-year-old male diagnosed with Mirizzi syndrome Type I. This case highlights the importance of early detection and appropriate management of Mirizzi syndrome, a rare yet clinically significant biliary condition. The diagnosis was confirmed through clinical presentation, laboratory findings, and imaging techniques. A comprehensive treatment approach, including ERCP with precut sphincterotomy, biliary plastic stent placement, and cholecystectomy, was employed to ensure optimal patient outcomes. This case contributes to the existing literature by emphasizing the necessity of prompt diagnosis and tailored management strategies to prevent complications. Gallstones are the leading cause of Mirizzi syndrome, but their formation and complications can be effectively prevented with proactive measures. Routine health check-ups are crucial for the early identification and management of gallstones, particularly for individuals with a family history or other risk factors. Furthermore, managing comorbidities such as diabetes and hypertension through medication adherence, dietary modifications, and lifestyle changes is key to reducing the risk of gallstones and preventing complications such as Mirizzi syndrome.
